# The Fibrillatory Wave Amplitude of ECG Decreases Over Time in Patients With Persistent AF: A Retrospective Cohort Study

**DOI:** 10.1111/anec.70161

**Published:** 2026-02-13

**Authors:** Jing Li, LingLi Wang, LeiLei Guo, ChunChang Qin

**Affiliations:** ^1^ Department of Cardiology The First Affiliated Hospital of Chongqing Medical University Chongqing China; ^2^ Department of Electrocardiogram The First Affiliated Hospital of Chongqing Medical University Chongqing China; ^3^ Department of Cardiology Jiang You People's Hospital Mianyang China

**Keywords:** atrial fibrillation, electrocardiography, radiofrequency ablation, the duration of atrial fibrillation history

## Abstract

**Background:**

A prolonged history of atrial fibrillation (AF) is associated with increased recurrence rates, and ablation is not recommended for AF lasting over 10 years. In some developing countries, patients may be unaware of when their AF began. Electrocardiogram (ECG) changes may correlate with AF duration, so this study aims to use ECG to estimate AF duration.

**Methods:**

This retrospective cohort study analyzed 998 patients with persistent AF. AF duration was the exposure variable, F‐wave amplitude and number of coarse F waves were outcomes. Correlations were evaluated using multiple linear regression, subgroup analysis, and smooth curve fitting.

**Results:**

Of the 998 participants, 50.2% were male, with a mean age of 73.0 years. The average F‐wave amplitude was 0.1 mV, and the average number of coarse F waves was 6.0. Longer AF duration was significantly associated with lower F‐wave amplitude and fewer coarse F waves. The highest quartile had significantly fewer coarse F waves (*β* = −4.94, 95% CI = −6.24 to −3.65, *p* < 0.001) and lower amplitude (*β* = −0.02, 95% CI = −0.03 to −0.02, *p* < 0.001) compared to the lowest quartile. A nonlinear inverse correlation was observed with cutoffs at 10.184 and 17.238 years. Additionally, left atrial dimension increased with longer AF duration.

**Conclusion:**

In patients with persistent AF, lower F‐wave amplitudes and fewer coarse F waves are associated with a longer history of AF.

## Graphical abstract

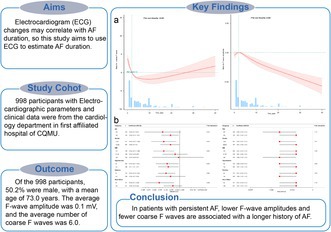

## 1 Introduction

Atrial fibrillation (AF) is the most common sustained arrhythmia and significantly increases the risk of death, stroke, heart failure, cognitive impairment and dementia (Chung et al. 2020; Madhavan et al. 2018). 1/3 of patients don't know they have AF and are underdiagnosed (Du et al. 2021), the severity of AF symptoms varies widely among individuals, and some patients may gradually tolerate the symptoms because they are nonspecific or mild, with about 1/4 of patients reporting themselves asymptomatic (Gibbs et al. 2021). Based on the current evidence, the guidelines recommend that opportunistic screening for AF by pulse palpation or ECG be considered at the time of the visit for those ≥ 65 years of age, and that systematic screening for AF by regular or continuous electrocardiographic monitoring be considered for those ≥ 70 years of age (Wang, Yang, et al. 2024).

Research has confirmed that the longer the duration of AF, the more significant the changes in cardiac function and structure (Allessie et al. 2002). The likelihood of restoring and maintaining sinus rhythm diminishes over time owing to the structural and electrical remodeling processes associated with long‐standing AF (Iwasaki et al. 2011). A growing body of research evidence supports the adoption of early rhythm control strategies in patients diagnosed with early AF or AF‐associated heart failure. Early rhythm control strategies are effective in reducing atrial remodeling; they can prevent AF‐related deaths, heart failure, and strokes in high‐risk populations. They have an important potential role in slowing the progression of AF and in reducing AF‐associated symptoms, and they should be applied to a wider range of patients with AF (Camm et al. 2022; Kim et al. 2021; Kirchhof et al. 2020). The duration of persistent AF directly impacts the effectiveness of ablation or other rhythm control strategies. Patients with longer durations of AF history have been shown to have worse outcomes with ablation (Stabile et al. 2019). The study shows that F‐wave amplitude is associated with AF termination during catheter ablation and clinical outcome after catheter ablation (Nault et al. 2009). The voltage of F‐wave is a useful predictor of procedural success of Maze procedure (Usui et al. 2022).

A prolonged history of AF is associated with increased recurrence rates, and ablation is not recommended for AF lasting over 10 years (Wang, Yang, et al. 2024). Therefore, selecting the suitable patients for radiofrequency ablation requires a comprehensive assessment of the duration of their AF history. However, in developing countries and remote areas, due to the lack of routine health screenings and medical recordings, some patients may be unaware of the duration of their AF history. Therefore, we expected to find a non‐invasive, simple indicator to provide a rough assessment of AF duration in patients who could not provide an exact history of AF.

In the body surface ECG of patients with persistent AF, P waves disappear and are replaced by AF waves. The amplitude of the F waves in the ECG can effectively reflect the electrical activity of atrial myocytes as well as the electrophysiological and structural changes in the atrium (Aysha and Hassan 1988). In patients with AF, the coarse F waves observed on the 12‐lead surface ECG are referred to as indicators of atrial vitality and contractility (Thurmann and Janney Jr. 1962). In clinical practice, we have observed that the characteristics of F waves in the V_1_ lead of the ECG are related to the duration of AF history; therefore, we hypothesize that there is a certain correlation between the amplitude of F waves in ECG and the number of coarse F waves with the duration of history in patients with persistent AF.

## 2 Methods

### 2.1 Data Source and Study Population

**FIGURE 1:**
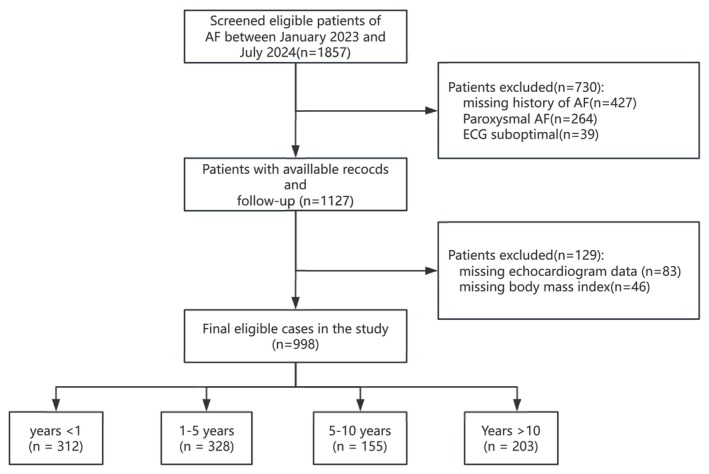
Flowchart of patient selection.

In this study, 1857 patients with AF were retrospectively random screened, at cardiology department in first affiliated hospital of CQMU, between January 2023 and July 2024. The inclusion criteria was: patients with persistent AF (Hindricks et al. 2021), patients were excluded from the study if they met any of the following exclusion criteria: (1) an uncertain history of AF, (2) Paroxysmal AF, (3) valvular AF, (4) patients taking antiarrhythmic drugs such as amiodarone, (5) the quality of the ECG is suboptimal due to: (1) inadequate signal quality (excessive baseline wander, muscle artifact, or poor lead contact) that precluded clear F‐wave identification in lead V_1_; (2) presence of a non‐conforming arrhythmia (typical atrial flutter or ventricular rate > 120 bpm preventing measurement within the QT interval due to T‐wave overlap); or (3) absence of a distinct, consistently measurable F‐wave morphology across cycles. We categorized the duration of AF history into clinically relevant intervals, dividing all patients into the following four groups: years < 1, 1–5 years, 5–10 years, years > 10. This categorization was chosen to distinguish between newly diagnosed, early‐stage, mid‐term, and long‐term AF history. After screening, the final cohort for further analysis included 998 individuals (Figure 1). Due to the retrospective nature of the study, informed consent was exempted. The study followed the Helsinki Declaration and adhered to the STROBE (Strengthening the Reporting of Observational Studies in Epidemiology) guidelines.

### 2.2 Diagnostic Criteria

The duration of AF was defined as the length of time that a patient has been experiencing AF (Hindricks et al. 2021; January et al. 2019). Hypertension was defined as either SBP ≥ 140 mmHg or DBP ≥ 90 mmHg, or having been previously diagnosed with hypertension and currently receiving antihypertensive treatment. Diabetes was defined as fasting blood glucose ≥ 7.0 mmol/L, or random blood glucose ≥ 11.1 mmol/L with typical symptoms, or glycated hemoglobin (HbA1c) ≥ 6.5%, or a clear previous diagnosis of diabetes and oral hypoglycemic drugs. Preexisting heart failure (Kawaji et al. 2022) was defined as either: (1) prior HF hospitalization, or (2) documented HF symptoms (NYHA class ≥ II) with structural heart disease or left ventricular ejection fraction (LVEF) < 40%. Preexisting HF with and without LVEF < 40% on transthoracic echocardiography were regarded as heart failure with reduced LVEF and preserved LVEF, respectively. According to Chinese body mass index (BMI) classification criteria (Jia et al. 2025), low weight: < 18.5 kg/m^2^, normal weight: 18.5–23.9 kg/m^2^, overweight: 24.0–27.9 kg/m^2^, obesity: ≥ 28.0 kg/m^2^.

### 2.3 Data Collection

The data of AF duration were collected from patients' previous visit records in our hospital's electronic medical record system, which had clearly recorded the specific AF duration. Age, gender, BMI, hypertension, diabetes, heart failure, and echocardiographic data were collected from the electronic medical record system of our hospital.

### 2.4 Electrocardiographic Evaluation

The 12‐lead surface ECGs of the patients (Beijing Medix, 25 mm/s speed and 1 mv/10 mm standard), the amplitude of the fibrillation wave was manually measured by two cardiologists. Although previous studies have employed methods for automatically analyzing the amplitude and morphology of AF waves (Aunes‐Jansson et al. 2013), our research utilizes high‐resolution manual measurements (Kawaji et al. 2022; Relander et al. 2024) to conduct a refined assessment of specific electrocardiogram features. This study employed an approach to measure the amplitude of F‐waves: 10 discrete F‐waves were selected during a period of slow ventricular rate to avoid superimposition of QRST and were measured manually from peak to trough in lead V_1_, using electronic calipers; F‐wave amplitude of V_1_ lead was the average voltage of all the measured F waves (Yin et al. 2017). Additionally, a 10‐s surface ECG from lead V_1_ the number of F‐waves with an amplitude ≥ 0.1 mv (coarse F‐waves) was recorded, with F‐waves ≥ 0.1 mv classified as coarse F‐waves and fine F‐waves defined by F‐wave < 0.1 mv (Thurmann and Janney Jr. 1962; Yamamoto et al. 2005) (The clinician can measure the F‐wave amplitude through the electronic caliper in the ECG reading system and can also estimate the number of small squares (the height of each small square is 0.1 mv) on the ECG drawing).

### 2.5 Echocardiographic Evaluation

From echocardiographic data (GE Vivid E95 and Philips IE33) to understand the size and function of the heart, LVEF, left ventricular diastole diameters (LVDD), left ventricular systolic diameters (LVSD), and left atrium diameter (LAD) were recorded (İçen et al. 2020).

### 2.6 Covariates

We evaluated possible confounding factors by utilizing our descriptive statistics and prior knowledge (İçen et al. 2020). We included the following variables: age, gender, BMI, hypertension, diabetes, heart failure, LVEF, LVDD, LVSD, LAD. The duration of AF serves as the exposure variable; the duration of AF was defined as the length of time that a patient has been experiencing AF (Hindricks et al. 2021; January et al. 2019).

### 2.7 Outcome Variable

The outcome variables were the F‐wave amplitude and the number of coarse F waves.

### 2.8 Statistical Analysis

The variables were divided into two categories, categorical and continuous. Categorical variables were shown as numbers and percentages, continuous variables as means (SD) or medians (IQR). Statistical tests such as ANOVA, Kruskal–Wallis, and chi‐squared tests were used to compare differences across groups. Our study examined the relationship between the duration of AF with the number of coarse F waves and F‐wave amplitude using an adjusted smooth curve for potential confounding factors. Linear regression models were used to examine the association between the duration of AF and the F‐wave amplitude and the number of coarse F waves, generating β and 95% confidence intervals (95% CI). In the multivariate linear regression analysis, three adjustment models were created: Model 1 was adjusted for age and gender. Model 2 was adjusted for model 1 plus BMI, hypertension, diabetes, heart failure, and LVEF. Model 3 was adjusted for model 2 plus LVDD, LVSD, LAD.

We used restricted cubic splines (RCS) with four knots (5th, 35th, 65th, 95th) to investigate the time‐response relationship between the duration of AF with the F‐wave amplitude and the number of coarse F waves, adjusting for confounding factors consistent with linear regression Model 3.

Additionally, we used a two‐piecewise linear regression model to examine the threshold association between the duration of AF with the F‐wave amplitude and the number of coarse F waves, adjusting for Model 3 variables. We also evaluated potential modifications of this relationship based on age, gender, BMI, hypertension, diabetes, heart failure categories using multivariate linear regression analysis with likelihood ratio tests for interactions.

The statistical analyses were conducted using R version 4.2.2 (http://www.R‐project.org, The R Foundation) and Free Statistics software version 1.9.2. Statistical significance was set at a two‐sided *p* value of < 0.05.

## 3 Results

### 3.1 Baseline Characteristics

Among 998 participants, of which 501 (50.2%) were male, with a mean age of 73.0 years. The baseline characteristics of enrolled participants are summarized in Table 1. This table shows average F‐wave amplitude was 0.1 mv, average number of coarse F waves was 6.0, mean LAD was 42.8 ± 8.4 mm. There were statistically significant differences between four groups in age, BMI, heart failure, LAD, F wave amplitude, coarse F wave (*p* < 0.05). As the duration of AF increases, the number of coarse F‐waves, LAD, age, and the prevalence of heart failure patients rises, the amplitude of F‐waves diminishes (*p* < 0.001), and BMI decreases (*p* = 0.011). Nevertheless, gender, hypertension, diabetes, LVEF, LVDD, and LVSD showed no significant variation (*p* > 0.05) (Table 1).

**TABLE 1:** Baseline characteristics of the patients with atrial fibrillation.

Variables	Total (*n* = 998)	Years < 1 (*n* = 312)	1–5 years (*n* = 328)	5–10 years (*n* = 155)	Years > 10 (*n* = 203)	*p*
Age (years)	73.0 ± 11.9	69.6 ± 13.1	73.6 ± 11.3	76.1 ± 10.5	75.0 ± 10.5	< 0.001
**Gender (%)**						0.065
Male	501 (50.2)	174 (55.8)	158 (48.2)	79 (51)	90 (44.3)	
Female	497 (49.8)	138 (44.2)	170 (51.8)	76 (49)	113 (55.7)	
BMI (kg/m^2^)	23.7 ± 3.9	24.2 ± 3.6	23.4 ± 3.6	23.9 ± 4.8	23.2 ± 4.1	0.011
**Hypertension (%)**						0.594
No	452 (45.3)	148 (47.4)	142 (43.3)	66 (42.6)	96 (47.3)	
Yes	546 (54.7)	164 (52.6)	186 (56.7)	89 (57.4)	107 (52.7)	
**Diabetes (%)**						0.955
No	712 (71.3)	226 (72.4)	232 (70.7)	111 (71.6)	143 (70.4)	
Yes	286 (28.7)	86 (27.6)	96 (29.3)	44 (28.4)	60 (29.6)	
**Heart failure**						< 0.001
No	371 (37.2)	155 (49.7)	114 (34.8)	48 (31)	54 (26.6)	
Yes	627 (62.8)	157 (50.3)	214 (65.2)	107 (69)	149 (73.4)	
LVEF (%)	57.3 ± 9.7	57.5 ± 9.2	57.4 ± 10.0	58.2 ± 9.1	56.0 ± 10.4	0.187
LVDD (mm)	49.5 ± 7.6	49.6 ± 7.1	49.4 ± 7.8	48.7 ± 7.3	50.3 ± 8.3	0.274
LVSD (mm)	34.8 ± 8.2	34.8 ± 7.8	34.7 ± 8.2	33.9 ± 7.8	35.7 ± 9.1	0.194
LAD (mm)	42.8 ± 8.4	40.4 ± 6.8	41.9 ± 7.3	43.8 ± 7.9	47.0 ± 10.6	< 0.001
F wave amplitude (mv)	0.1 ± 0.0	0.1 ± 0.0	0.1 ± 0.0	0.1 ± 0.0	0.1 ± 0.0	< 0.001
Coarse F wave (n)	6.0 (0.0, 15.0)	15.0 (8.0, 22.0)	8.0 (2.0, 15.0)	3.0 (0.0, 9.0)	0.0 (0.0, 0.0)	< 0.001

Abbreviations: BMI, body mass index; LAD, left atrial diameter; LVEF, left ventricular ejection fraction; LVDD, Left ventricular diastolic diameter; LVSD, left ventricular systolic diameter.

### 3.2 Univariate Analysis

The results of univariate analysis are shown in Table 2. The results of univariate analysis showed that age, gender, the duration of AF, diabetes, heart failure, LAD, and F‐wave amplitude were correlated with the number of coarse F waves. And that age, gender, the number of coarse F waves, heart failure, LAD were correlated with F‐wave amplitude.

**TABLE 2:** Univariate analysis of patients with atrial fibrillation.

Item	Coeff. (95% CI)	*p* (t‐test)	*p* (F‐test)	Item	Coeff. (95% CI)	*p* (t‐test)	*p* (F‐test)
Gender: female vs. male	−1.54 (−2.75, −0.33)	0.013	0.013	Time (cont. var.)	0 (0,0)	< 0.001	< 0.001
Age (cont. var.)	−0.13 (−0.18, −0.08)	< 0.001	< 0.001	Gender: female vs. male	−0.01 (−0.01,0)	0.043	0.043
Time (cont. var.)	−0.63 (−0.71, −0.56)	< 0.001	< 0.001	Age (cont. var.)	0 (0,0)	< 0.001	< 0.001
BMI (cont. var.)	−0.04 (−0.19,0.12)	0.633	0.633	BMI (cont. var.)	0 (0,0)	0.602	0.602
Hypertension: yes vs. no	−0.71 (−1.93,0.51)	0.253	0.253	Hypertension: yes vs. no	0 (−0.01,0.01)	0.865	0.865
Diabetes: yes vs. no	−1.77 (−3.11, −0.44)	0.009	0.009	Diabetes: yes vs. no	0 (−0.01,0)	0.203	0.203
Heart failure: yes vs. no	−1.59 (−2.84, −0.33)	0.013	0.013	Heart failure: yes vs. no	−0.01 (−0.01,0)	0.046	0.046
LVEF (cont. var.)	0.06 (0,0.12)	0.058	0.058	LVEF (cont. var.)	0 (0,0)	0.666	0.666
LVDD (cont. var.)	−0.03 (−0.11,0.05)	0.493	0.493	LVDD (cont. var.)	0 (0,0)	0.848	0.848
LVSD (cont. var.)	−0.03 (−0.1,0.04)	0.407	0.407	LVSD (cont. var.)	0 (0,0)	0.491	0.491
LAD (cont. var.)	−0.17 (−0.25, −0.1)	< 0.001	< 0.001	LAD (cont. var.)	0 (0,0)	< 0.001	< 0.001
F amplitude (cont. var.)	155.57 (147.48,163.65)	< 0.001	< 0.001	Coarse F wave (cont. var.)	0 (0,0)	< 0.001	< 0.001

Abbreviations: BMI, body mass index; LAD, left atrial diameter; LVEF, left ventricular ejection fraction; LVDD, left ventricular diastolic diameter; LVSD, left ventricular systolic diameter.

### 3.3 Relationship Between the Duration of AF With the Number of Coarse F Waves and F‐Wave Amplitude

Using a linear regression model with multiple variables, the duration of AF was divided into quartile, and after controlling for potential confounders, a notable negative relationship was observed between the duration of AF with the number of coarse F waves and F‐wave amplitude prevalence, when analyzed as continuous variables (per 10‐year increment), both the number of coarse F waves and F‐wave amplitude showed a significant inverse association with longer AF duration (*β* = −1.1, 95% CI = −0.17 to −0.04, *p* = 0.002; *β* = −0.01, 95% CI = −0.02 to −0.01, *p* < 0.001). Those in the highest quartile (Q4) had fewer the number of coarse F waves (*β* = −4.94, 95% CI = −6.24 to −3.65, *p* < 0.001) (Table 3, model 3) and lower F‐wave amplitude (*β* = −0.02, 95% CI = −0.03 to −0.02, *p* < 0.001) (Table 4, model 3) compared with those in the lowest quartile (Q1) after adjustment for potential confounding factors.

**TABLE 3:** Multivariable linear regression analyses of Coarse F wave and the duration of atrial fibrillation history.

	Crude mode		Model 1		Model 2		Model 3	
	β (95% CI)	*p*	β (95% CI)	*p*	β (95% CI)	*p*	β (95% CI)	*p*
Time (per 10 years)	−6.3 (−0.71 ~ −0.56)	< 0.001	−6.2 (−0.69 ~ −0.54)	< 0.001	−6.2 (−0.7 ~ −0.54)	< 0.001	−1.1 (−0.17 ~ −0.04)	0.002
Q_1_ (< 1)	0 (Ref)		0 (Ref)		0 (Ref)		0 (Ref)	
Q_2_ (1–5)	−6.01 (−7.27 ~ −4.74)	< 0.001	−5.8 (−7.08 ~ −4.53)	< 0.001	−6 (−7.27 ~ −4.73)	< 0.001	−3.46 (−4.42 ~ −2.51)	< 0.001
Q_3_ (5–10)	−10.02 (−11.59 ~ −8.45)	< 0.001	−9.73 (−11.33 ~ −8.14)	< 0.001	−9.91 (−11.5 ~ −8.32)	< 0.001	−5.15 (−6.37 ~ −3.92)	< 0.001
Q_4_ (> 10)	−14.76 (−16.2 ~ −13.32)	< 0.001	−14.48 (−15.94 ~ −13.02)	< 0.001	−14.66 (−16.13 ~ −13.2)	< 0.001	−4.94 (−6.24 ~ −3.65)	< 0.001
*p* for trend		< 0.001		< 0.001		< 0.001		< 0.001

*Note:* Time: the duration of AF history. Crude model was adjusted for none. Model 1 was adjusted for age and gender. Model 2 was adjusted for model 1 plus BMI, Hypertension, Diabetes, Heart failure and LVEF. Model 3 was adjusted for model 1plus model 2 plus LVDD, LVSD, LAD and F amplitude.

**TABLE 4:** Multivariable linear regression analyses of F wave amplitude and the duration of atrial fibrillation history.

	Crude mode		Model 1		Model 2		Model 3	
	β (95% CI)	*p*	β (95% CI)	*p*	β (95% CI)	*p*	β (95% CI)	*p*
Time (per 10 years)	−0.04 (−0.04 ~ −0.03)	< 0.001	−0.04 (−0.04 ~ −0.03)	< 0.001	−0.04 (−0.04 ~ −0.03)	< 0.001	−0.01 (−0.02 ~ −0.01)	< 0.001
Q_1_ (< 1)	0 (Ref)		0 (Ref)		0 (Ref)		0 (Ref)	
Q_2_ (1–5)	−0.02 (−0.03 ~ −0.01)	< 0.001	−0.02 (−0.02 ~ −0.01)	< 0.001	−0.02 (−0.03 ~ −0.01)	< 0.001	0 (0 ~ 0.01)	0.518
Q_3_ (5–10)	−0.04 (−0.04 ~ −0.03)	< 0.001	−0.04 (−0.04 ~ −0.03)	< 0.001	−0.04 (−0.04 ~ −0.03)	< 0.001	0 (−0.01 ~ 0)	0.626
Q_4_ (> 10)	−0.07 (−0.08 ~ −0.07)	< 0.001	−0.07 (−0.08 ~ −0.06)	< 0.001	−0.07 (−0.08 ~ −0.07)	< 0.001	−0.02 (−0.03 ~ −0.02)	< 0.001
*p* for trend		< 0.001		< 0.001		< 0.001		< 0.001

*Note:* Time: the duration of AF history. Crude model was adjusted for none. Model 1 was adjusted for age and gender. Model 2 was adjusted for model 1 plus BMI, Hypertension, Diabetes, Heart failure and LVEF. Model 3 was adjusted for model 1plus model 2 plus LVDD, LVSD, LAD and Coarse F wave.

**FIGURE 2:**
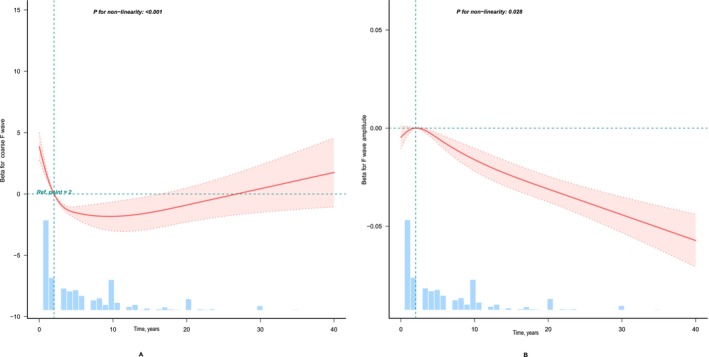
Nonlinear relationship between the duration of AF with the number of coarse F waves and F‐wave amplitude. Adjustment factors included age, sex, body mass index (BMI), hypertension, diabetes, heart failure, left ventricular ejection fraction (LVEF); Left ventricular diastolic diameter (LVDD); Left ventricular systolic diameter (LVDS); Left atrial diameter (LAD). The red line and pink area represent the estimated values and their corresponding 95% confidence intervals, respectively. Panel (A) shows the relationship between the duration of AF with the number of coarse F waves, while (B) presents the relationship between the duration of AF and F‐wave amplitude.

Notably, as shown in Figure 2, a nonlinear relationship (*p* for nonlinearity < 0.001 Figure 2A) was observed between the duration of AF and the number of coarse F waves. The relationship between the duration of AF and F‐wave amplitude, a non‐linear association, is observed (*p* for nonlinear = 0.028 Figure 2B). Using threshold analysis, when the duration of AF is less than 10.184 years, it had a *β* of −1.327 (95% CI: −1.515 to −1.14, *p* < 0.001) and when the duration of AF is less than 17.238 years, it had a *β* of −0.005 (95% CI: −0.006 to −0.005, *p* < 0.001) (Table 5). This indicates that when the duration of AF is less than 10.184 years, for each additional year, the number of coarse F‐waves decreases by 1.327; when the duration of AF is less than 17.238 years, for each additional year, the F‐wave amplitude decreases by 0.005 mV (*p* < 0.001).

**TABLE 5:** Threshold effect analysis of the relationship of Coarse F wave/F wave amplitude and duration of atrial fibrillation history.

	Coarse F wave	
	β (95% CI)	*p*
Time < 10.184	−1.327 (−1.515 ~ −1.14)	< 0.001
Time ≥ 10.184	−0.035 (−0.065 ~ 0.004)	0.026
Likelihood ratio test		< 0.001

	F wave amplitude	
	β (95% CI)	*p*
Time < 17.238	−0.005 (−0.006 ~ −0.005)	< 0.001
Time ≥ 17.238	0 (−0.002 ~ 0.001)	0.655
Likelihood ratio test		< 0.001

*Note:* Time: the duration of AF history. Adjusted for age, gender, BMI, hypertension, diabetes, heart failure and LVEF, LVDD, LVSD, LAD. 100% of the data is displayed.

### 3.4 Subgroup Analyses

**FIGURE 3:**
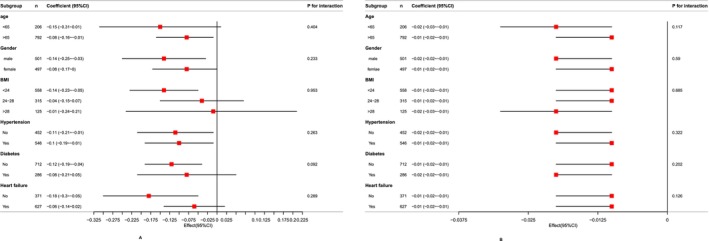
Subgroup analysis of relationship between the duration of AF with the number of coarse F waves and F‐wave amplitude. The correlation coefficient (*β*) were adjusted for left ventricular ejection fraction (LVEF); Left ventricular diastolic diameter (LVDD); Left ventricular systolic diameter (LVDS); Left atrial diameter (LAD). Panel (A) shows the relationship between the duration of AF with the number of coarse F waves, while (B) presents the relationship between the duration of AF and F‐wave amplitude.

To find out whether subgroups had different effects on the relationship between the duration of AF with the number of coarse F waves and F‐wave amplitude, stratified analyses were performed. Subgroup analyses across various categories showed no notable interactions within any subgroup based on age, gender, BMI, hypertension, diabetes, heart failure status (*p* < 0 0.05) (Figure 3).

## 4 Discussion

In this retrospective cohort study, we found a negative correlation between the duration of AF history with F wave amplitude on V_1_ lead ECG and the number of coarse F waves in 10s of V_1_ lead ECG, even after adjusting for confounding factors. We observed a nonlinear relationship between the duration of AF with the number of coarse F waves, with cutoff values of 10.184 years. We observed a nonlinear association between the duration of AF with F‐wave amplitude, with cutoff values of 17.238 years. Additionally, LAD increased with longer AF duration.

Our research found that as the duration of AF prolongs, the amplitude of F‐waves and the number of coarse F‐waves gradually decrease, when the duration of AF is less than 10.184 years, for each additional year, the number of coarse F‐waves decreases by 1.327, when the duration of AF is less than 17.238 years, for each additional year, the F‐wave amplitude decreases by 0.005 mV (*p* < 0.001). Although the predictive uncertainty increases in the long‐history range (> 10 years) due to a reduced number of available cases, the model reveals a significant nonlinear association between recurrence risk and AF history across the entire spectrum (*p* for nonlinearity < 0.001). This suggests that among ablation‐eligible patients, even for the subgroup with a longer history, the risk trend remains consistent with the overall model, while prognostic assessment for such individuals must fully account for the higher uncertainty. Our findings align with earlier research conclusions: prolonged duration of atrial fibrillation is associated with a reduction in amplitude (Nault et al. 2009), and subsequent studies have also observed that as the duration of atrial fibrillation increases, the amplitude of the F‐wave shows a decreasing trend (Pourafkari et al. 2018). Patients with longer AF history had a greater prevalence of structural heart disease and atrial cardiomyopathy and were more likely to be in AF at baseline (Blomström‐Lundqvist et al. 2020). Studies have shown that the average amplitude of the F wave in the V_1_ lead of surface ECG holds significant reference value for diagnosing recurrence in patients with persistent AF after radiofrequency ablation (Wang, Guo, et al. 2024). The longer the duration of AF, the more pronounced the changes in cardiac function and structure, leading to a higher recurrence rate of AF post‐ablation (Sultan et al. 2017).

The morphology of the surface ECG F wave reflects atrial electrical activity (Haissaguerre 2024; Hsu et al. 2008). Fibrillatory wave amplitude on surface ECG is dependent on the magnitude of the underlying voltage, which is related to the magnitude of remaining viable (Nault et al. 2009). Fibrosis is a major contributor to AF maintenance (Jalife 2014), the longer the duration of AF, the more severe the degree of atrial fibrosis; as the degree of atrial fibrosis increases, the amplitude of the F wave decreases (Yamamoto et al. 2005) (Marrouche et al. 2014). Atrial fibrillation persists for more than 10 years, and the EGC shows extremely fine F‐waves that are nearly straight. This explains the L‐shaped correlation between the duration of AF observed in this study and the amplitude of F‐waves and the number of coarse F‐waves. The amplitude of F‐waves and the number of coarse F‐waves are associated with atrial electrical remodeling, which reduces the length of the atrial wave and promotes the occurrence and maintenance of AF (Allessie et al. 2002). The greater the number of wavelets, the smaller the composite vector across different regions of the atrium, resulting in finer F‐waves and smaller amplitude (Rahme et al. 1999), this may explain why chronic AF is characterized by a decrease in F‐waves amplitude and a reduction in the number of coarse F‐waves compared to paroxysmal AF. Yin et al. (2017) demonstrated a significant inverse correlation between the amplitude of fibrillatory wave and the size of low voltage area in the LA. The persistent episodes of AF can lead to electrophysiological alterations in the atria. The fine F‐wave is associated with fragmented activation patterns and conduction delays (Saksena et al. 1999). The amplitude of the F‐wave is influenced by several factors, such as atrial hypertrophy or dilation, atrial myocyte degeneration, atrial fibrosis, or the duration of AF (Aysha and Hassan 1988; Morganroth et al. 1979; Sanfilippo et al. 1990). Compared to normal myocardium, myocardial fibrosis results in prolonged depolarization times and a reduction in the number of myocardial cells. Additionally, it causes uneven electrical conduction, which may lead to localized conduction reentry or block. Ultimately, it will impact the morphology of the surface ECG.

Previous studies have analyzed the echocardiographic, Sanfilippo et al.'s (1990) results indicate that atrial enlargement can occur as a consequence of AF, one study revealed larger LA size is associated with fine AF amplitude (Nault et al. 2009). However, in various studies, the correlation between LAD and F‐wave amplitude has shown inconstantly (Bollmann et al. 2001; Mutlu et al. 2003), prior studies have found atrial enlargement in both fine (Yamamoto et al. 2005) and coarse (Thurmann and Janney Jr. 1962) F‐wave groups. We observed that with the prolonged duration of AF, there is a gradual enlargement of the left atrial dimension. Actually, Chen et al. recently reported similar results (Chen et al. 2021), and our findings are consistent with Blomström‐Lundqvist et al.'s (2020) results. Furthermore, Petersen et al. showed that significant differences in LA dimensions between patients with chronic AF of short and long durations and observed a significant increase in LA dimensions after 6 months of follow‐up in both groups, suggesting that AF contributes to atrial enlargement over time (Petersen et al. 1987), our findings also are supported by Petersen et al.'s results.

This study presents several notable advantages. First, this study is the first, to our knowledge, to used F waves and the number of coarse F waves to estimate the duration of AF, moreover, in clinical context, the number of coarse F‐waves is more readily obtainable, requiring less time. Second, this metric offers a straightforward, non‐invasive, and rapid approach to estimate a patient's duration of AF, particularly suitable for developing countries and remote areas and resource‐limited settings, for the preliminary assessment of the duration of AF history. Third, the success of radiofrequency ablation is related to duration of AF, F‐waves and degree of LA Enlargement (Escribano et al. 2022; Naji et al. 2024; Schnabel et al. 2010), the duration between the clinical diagnosis of AF and the initial catheter ablation is an emerging clinical predictor of ablation success (Chew et al. 2022). Kainuma et al. highlight the fact that ablation therapy may not be the optimal treatment option for all AF patients. Therefore, evaluating duration of AF noninvasively through the amplitude of F waves and the number of coarse F waves on lead V_1_ of ECG would be clinically useful if patients likely to respond could be identified and selected for ablation while other patients not likely to respond to ablation could be counseled against this procedure.

## 5 Limitations

Several limitations of this study warrant consideration. First, as a single‐center retrospective investigation, the potential for selection bias may compromise the accuracy of our findings. Consequently, there is a need to refine the research protocol and conduct multicenter studies to validate the research conclusions. Second, due to the missing data on BNP and routine blood tests and other variables that can possibly effect F wave amplitude in the ECG (such as COPD history, presence of pericardial effusion etc.), insufficient collection of covariate data; in the future, the scope of covariate data collection can be expanded to further analyze its correlation. Third, this study didn't explore the confounding factor that similar F‐wave patterns occur in patients with paroxysmal AF, further verification in combination with imaging or biomarkers is needed in the future.

## 6 Conclusions

In summary, we found a nonlinear inverse correlation between the duration of AF with the number of coarse F waves and F‐wave amplitude in patients with persistent AF. Specifically, lower F‐wave amplitudes and fewer coarse F waves are associated with a longer history of AF.

## Author Contributions

ChunChang Qin designed the research study; Jing Li and LingLi Wang made contributions to the data gathering, LeiLei Guo and Jing Li analyzed the data, and Jing Li and LingLi Wang wrote the manuscript. All authors contributed to editorial changes in the manuscript. All authors read and approved the final manuscript. All authors have participated sufficiently in the work and agreed to be accountable for all aspects of the work. All authors reviewed and approved the final version of the manuscript. All authors take responsibility for all aspects of the reliability and freedom from bias of the data presented and their discussed interpretation.

## Funding

This research was funded by the National Natural Science Foundation of China (to ChunChang Qin No. 82070523); the Chongqing Science and Technology Bureau (to ChunChang Qin NO. cstc2019jscx‐msxmX0307); and the Chongqing Health Commission (to ChunChang Qin No. 2020msxm113).

## Disclosure

The authors have nothing to report.

## Ethics Statement

The study followed the Helsinki Declaration. The study was approved by the first affiliated hospital of Chongqing Medical University Institutional Review Board, number is 2024‐515‐01, and date 18.12.2024.

## Consent

Due to the retrospective nature of the study, informed consent was exempted.

## Conflicts of Interest

The authors declare no conflicts of interest.

## Data Availability

The authors of this paper have provided the original data to support the conclusions of this paper without unnecessary reservations.

## References

[anec70161-bib-0001] Allessie, M. , J. Ausma , and U. Schotten . 2002. “Electrical, Contractile and Structural Remodeling During Atrial Fibrillation.” Cardiovascular Research 54, no. 2: 230–246. 10.1016/s0008-6363(02)00258-4. 12062329

[anec70161-bib-0002] Aunes‐Jansson, M. , N. Edvardsson , M. Stridh , L. Sörnmo , L. Frison , and A. Berggren . 2013. “Decrease of the Atrial Fibrillatory Rate, Increased Organization of the Atrial Rhythm and Termination of Atrial Fibrillation by AZD7009.” Journal of Electrocardiology 46, no. 1: 29–35. 10.1016/j.jelectrocard.2012.09.002. 23219385

[anec70161-bib-0003] Aysha, M. H. , and A. S. Hassan . 1988. “Diagnostic Importance of Fibrillatory Wave Amplitude: A Clue to Echocardiographic Left Atrial Size and Etiology of Atrial Fibrillation.” Journal of Electrocardiology 21, no. 3: 247–251. 10.1016/0022-0736(88)90099-4. 2971749

[anec70161-bib-0004] Blomström‐Lundqvist, C. , N. Marrouche , S. Connolly , et al. 2020. “Efficacy and Safety of Dronedarone by Atrial Fibrillation History Duration: Insights From the ATHENA Study.” Clinical Cardiology 43, no. 12: 1469–1477. 10.1002/clc.23463. 33080088 PMC7724236

[anec70161-bib-0005] Bollmann, A. , K. Binias , F. Grothues , et al. 2001. “Left Atrial Appendage Flow in Nonrheumatic Atrial Fibrillation: Relationship With Pulmonary Venous Flow and ECG Fibrillatory Wave Amplitude.” Chest 119, no. 2: 485–492. 10.1378/chest.119.2.485. 11171727

[anec70161-bib-0006] Camm, A. J. , G. V. Naccarelli , S. Mittal , et al. 2022. “The Increasing Role of Rhythm Control in Patients With Atrial Fibrillation: JACC State‐of‐the‐Art Review.” Journal of the American College of Cardiology 79, no. 19: 1932–1948. 10.1016/j.jacc.2022.03.337. 35550691

[anec70161-bib-0007] Chen, Y. C. , A. Voskoboinik , A. Gerche , T. H. Marwick , and J. R. McMullen . 2021. “Prevention of Pathological Atrial Remodeling and Atrial Fibrillation: JACC State‐of‐the‐Art Review.” Journal of the American College of Cardiology 77, no. 22: 2846–2864. 10.1016/j.jacc.2021.04.012. 34082914

[anec70161-bib-0008] Chew, D. S. , K. A. Jones , Z. Loring , et al. 2022. “Diagnosis‐to‐Ablation Time Predicts Recurrent Atrial Fibrillation and Rehospitalization Following Catheter Ablation.” Heart Rhythm O2 3, no. 1: 23–31. 10.1016/j.hroo.2021.11.012. 35243432 PMC8859793

[anec70161-bib-0009] Chung, M. K. , M. Refaat , W. K. Shen , et al. 2020. “Atrial Fibrillation: JACC Council Perspectives.” Journal of the American College of Cardiology 75, no. 14: 1689–1713. 10.1016/j.jacc.2020.02.025. 32273035

[anec70161-bib-0010] Du, X. , L. Guo , S. Xia , et al. 2021. “Atrial Fibrillation Prevalence, Awareness and Management in a Nationwide Survey of Adults in China.” Heart 107, no. 7: 535–541. 10.1136/heartjnl-2020-317915. 33509976 PMC7958113

[anec70161-bib-0011] Escribano, P. , J. Ródenas , M. García , et al. 2022. “Preoperative Prediction of Catheter Ablation Outcome in Persistent Atrial Fibrillation Patients Through Spectral Organization Analysis of the Surface Fibrillatory Waves.” Journal of Personalized Medicine 12, no. 10: 721. 10.3390/jpm12101721. 36294860 PMC9604697

[anec70161-bib-0012] Gibbs, H. , B. Freedman , M. Rosenqvist , et al. 2021. “Clinical Outcomes in Asymptomatic and Symptomatic Atrial Fibrillation Presentations in GARFIELD‐AF: Implications for AF Screening.” American Journal of Medicine 134, no. 7: 893–901.e811. 10.1016/j.amjmed.2021.01.017. 33607088

[anec70161-bib-0013] Haissaguerre, M. 2024. “Spontaneous Initiation of Atrial Fibrillation by Ectopic Beats Originating in the Pulmonary Veins.” Heart Rhythm 21, no. 6: 713–714. 10.1016/j.hrthm.2024.03.046. 38816145

[anec70161-bib-0014] Hindricks, G. , T. Potpara , N. Dagres , et al. 2021. “2020 ESC Guidelines for the Diagnosis and Management of Atrial Fibrillation Developed in Collaboration With the European Association for Cardio‐Thoracic Surgery (EACTS): The Task Force for the Diagnosis and Management of Atrial Fibrillation of the European Society of Cardiology (ESC) Developed With the Special Contribution of the European Heart Rhythm Association (EHRA) of the ESC.” European Heart Journal 42, no. 5: 373–498. 10.1093/eurheartj/ehaa612. 32860505

[anec70161-bib-0015] Hsu, N. W. , Y. J. Lin , C. T. Tai , et al. 2008. “Frequency Analysis of the Fibrillatory Activity From Surface ECG Lead V1 and Intracardiac Recordings: Implications for Mapping of AF.” Europace 10, no. 4: 438–443. 10.1093/europace/eun045. 18319264

[anec70161-bib-0016] İçen, Y. K. , H. Koca , H. E. Sümbül , et al. 2020. “Relationship Between Coarse F Waves and Thromboembolic Events in Patients With Permanent Atrial Fibrillation.” Journal of Arrhythmia 36, no. 6: 1025–1031. 10.1002/joa3.12430. 33335620 PMC7733569

[anec70161-bib-0017] Iwasaki, Y. K. , K. Nishida , T. Kato , and S. Nattel . 2011. “Atrial Fibrillation Pathophysiology: Implications for Management.” Circulation 124, no. 20: 2264–2274. 10.1161/circulationaha.111.019893. 22083148

[anec70161-bib-0018] Jalife, J. 2014. “Novel Upstream Approaches to Prevent Atrial Fibrillation Perpetuation.” Cardiology Clinics 32, no. 4: 637–650. 10.1016/j.ccl.2014.07.004. 25443242

[anec70161-bib-0019] January, C. T. , L. S. Wann , H. Calkins , et al. 2019. “2019 AHA/ACC/HRS Focused Update of the 2014 AHA/ACC/HRS Guideline for the Management of Patients With Atrial Fibrillation: A Report of the American College of Cardiology/American Heart Association Task Force on Clinical Practice Guidelines and the Heart Rhythm Society.” Journal of the American College of Cardiology 74, no. 1: 104–132. 10.1016/j.jacc.2019.01.011. 30703431

[anec70161-bib-0020] Jia, X. , C. Hu , Y. Xu , et al. 2025. “Revisiting Obesity Thresholds for Cardiovascular Disease and Mortality Risk in Chinese Adults: Age‐ and Gender‐Specific Insights.” Cell Reports Medicine 6, no. 9: 102309. 10.1016/j.xcrm.2025.102309. 40858104 PMC12490207

[anec70161-bib-0021] Kawaji, T. , H. Ogawa , Y. Hamatani , et al. 2022. “Fine Fibrillatory Wave as a Risk Factor for Heart Failure Events in Patients With Atrial Fibrillation: The Fushimi Atrial Fibrillation (AF) Registry.” Journal of the American Heart Association 11, no. 7: e024341. 10.1161/jaha.121.024341. 35322687 PMC9075419

[anec70161-bib-0022] Kim, D. , P. S. Yang , S. C. You , et al. 2021. “Treatment Timing and the Effects of Rhythm Control Strategy in Patients With Atrial Fibrillation: Nationwide Cohort Study.” BMJ 373: n991. 10.1136/bmj.n991. 33975876 PMC8111568

[anec70161-bib-0023] Kirchhof, P. , A. J. Camm , A. Goette , et al. 2020. “Early Rhythm‐Control Therapy in Patients With Atrial Fibrillation.” New England Journal of Medicine 383, no. 14: 1305–1316. 10.1056/NEJMoa2019422. 32865375

[anec70161-bib-0024] Madhavan, M. , J. Graff‐Radford , J. P. Piccini , and B. J. Gersh . 2018. “Cognitive Dysfunction in Atrial Fibrillation.” Nature Reviews. Cardiology 15, no. 12: 744–756. 10.1038/s41569-018-0075-z. 30275499

[anec70161-bib-0025] Marrouche, N. F. , D. Wilber , G. Hindricks , et al. 2014. “Association of Atrial Tissue Fibrosis Identified by Delayed Enhancement MRI and Atrial Fibrillation Catheter Ablation: The DECAAF Study.” JAMA 311, no. 5: 498–506. 10.1001/jama.2014.3. 24496537

[anec70161-bib-0026] Morganroth, J. , L. N. Horowitz , M. E. Josephson , and J. A. Kastor . 1979. “Relationship of Atrial Fibrillatory Wave Amplitude to Left Atrial Size and Etiology of Heart Disease. An Old Generalization Re‐Examined.” American Heart Journal 97, no. 2: 184–186. 10.1016/0002-8703(79)90354-5. 153707

[anec70161-bib-0027] Mutlu, B. , M. Karabulut , E. Eroglu , et al. 2003. “Fibrillatory Wave Amplitude as a Marker of Left Atrial and Left Atrial Appendage Function, and a Predictor of Thromboembolic Risk in Patients With Rheumatic Mitral Stenosis.” International Journal of Cardiology 91, no. 2–3: 179–186. 10.1016/s0167-5273(03)00024-x. 14559128

[anec70161-bib-0028] Naji, F. H. , J. Alatic , I. Balevski , and D. Suran . 2024. “Left Atrial Volume Index Predicts Atrial Fibrillation Recurrence After Catheter Ablation Only in Obese Patients‐Brief Report.” Diagnostics (Basel) 14, no. 14: 570. 10.3390/diagnostics14141570. 39061707 PMC11275257

[anec70161-bib-0029] Nault, I. , N. Lellouche , S. Matsuo , et al. 2009. “Clinical Value of Fibrillatory Wave Amplitude on Surface ECG in Patients With Persistent Atrial Fibrillation.” Journal of Interventional Cardiac Electrophysiology 26, no. 1: 11–19. 10.1007/s10840-009-9398-3. 19404588

[anec70161-bib-0030] Petersen, P. , J. Kastrup , K. Brinch , J. Godtfredsen , and G. Boysen . 1987. “Relation Between Left Atrial Dimension and Duration of Atrial Fibrillation.” American Journal of Cardiology 60, no. 4: 382–384. 10.1016/0002-9149(87)90253-0. 2956853

[anec70161-bib-0031] Pourafkari, L. , A. Baghbani‐Oskouei , N. Aslanabadi , et al. 2018. “Fine Versus Coarse Atrial Fibrillation in Rheumatic Mitral Stenosis: The Impact of Aging and the Clinical Significance.” Annals of Noninvasive Electrocardiology 23, no. 4: e12540. 10.1111/anec.12540. 29504703 PMC6931713

[anec70161-bib-0032] Rahme, M. M. , B. Cotter , E. Leistad , et al. 1999. “Persistence of Atrial Fibrillation After Its Induction‐Importance of the Duration and Dispersion of Atrial Refractoriness and Electrical Remodeling.” Journal of Cardiovascular Pharmacology and Therapeutics 4, no. 2: 113–120. 10.1177/107424849900400206. 10684530

[anec70161-bib-0033] Relander, A. , S. Jaakkola , H. Virri , et al. 2024. “Fibrillatory Wave Amplitude and Thromboembolic Risk in Non‐Anticoagulated Patients With Atrial Fibrillation.” Annals of Medicine 56, no. 1: 2317362. 10.1080/07853890.2024.2317362. 38350436 PMC10866044

[anec70161-bib-0034] Saksena, S. , I. Giorgberidze , R. Mehra , et al. 1999. “Electrophysiology and Endocardial Mapping of Induced Atrial Fibrillation in Patients With Spontaneous Atrial Fibrillation.” American Journal of Cardiology 83, no. 2: 187–193. 10.1016/s0002-9149(98)00822-4. 10073819

[anec70161-bib-0035] Sanfilippo, A. J. , V. M. Abascal , M. Sheehan , et al. 1990. “Atrial Enlargement as a Consequence of Atrial Fibrillation. A Prospective Echocardiographic Study.” Circulation 82, no. 3: 792–797. 10.1161/01.cir.82.3.792. 2144217

[anec70161-bib-0036] Schnabel, R. B. , M. G. Larson , J. F. Yamamoto , et al. 2010. “Relations of Biomarkers of Distinct Pathophysiological Pathways and Atrial Fibrillation Incidence in the Community.” Circulation 121, no. 2: 200–207. 10.1161/circulationaha.109.882241. 20048208 PMC3224826

[anec70161-bib-0037] Stabile, G. , S. A. Trines , and C. Blomström Lundqvist . 2019. “Atrial Fibrillation History Impact on Catheter Ablation Outcome.” Pacing and Clinical Electrophysiology 42, no. 6: 759. 10.1111/pace.13645. 30828818

[anec70161-bib-0038] Sultan, A. , J. Lüker , D. Andresen , et al. 2017. “Predictors of Atrial Fibrillation Recurrence After Catheter Ablation: Data From the German Ablation Registry.” Scientific Reports 7, no. 1: 16678. 10.1038/s41598-017-16938-6. 29192223 PMC5709464

[anec70161-bib-0039] Thurmann, M. , and J. G. Janney Jr. 1962. “The Diagnostic Importance of Fibrillatory Wave Size.” Circulation 25: 991–994. 10.1161/01.cir.25.6.991. 13921116

[anec70161-bib-0040] Usui, R. , M. Mutsuga , Y. Narita , et al. 2022. “Higher F‐Wave Frequency Associates With Poor Procedural Success Rate After Maze Procedure.” General Thoracic and Cardiovascular Surgery 70, no. 12: 997–1004. 10.1007/s11748-022-01836-0. 35771344

[anec70161-bib-0041] Wang, L. , G. Yang , C. Cui , et al. 2024. “The Feasibility of Atrial Fibrillatory Wave Amplitude in Predicting Ablation Outcomes in Persistent Atrial Fibrillation.” Journal of Electrocardiology 86: 153766. 10.1016/j.jelectrocard.2024.153766. 39197227

[anec70161-bib-0042] Wang, Y. , Y. Guo , M. Qin , et al. 2024. “2024 Chinese Expert Consensus Guidelines on the Diagnosis and Treatment of Atrial Fibrillation in the Elderly, Endorsed by Geriatric Society of Chinese Medical Association (Cardiovascular Group) and Chinese Society of Geriatric Health Medicine (Cardiovascular Branch): Executive Summary.” Thrombosis and Haemostasis 124, no. 10: 897–911. 10.1055/a-2325-5923. 38744425 PMC11436293

[anec70161-bib-0043] Yamamoto, S. , M. Suwa , T. Ito , et al. 2005. “Comparison of Frequency of Thromboembolic Events and Echocardiographic Findings in Patients With Chronic Nonvalvular Atrial Fibrillation and Coarse Versus Fine Electrocardiographic Fibrillatory Waves.” American Journal of Cardiology 96, no. 3: 408–411. 10.1016/j.amjcard.2005.03.087. 16054469

[anec70161-bib-0044] Yin, R. , Y. Fu , Z. Yang , B. Li , J. Pen , and Z. Zheng . 2017. “Fibrillatory Wave Amplitude on Transesophageal ECG as a Marker of Left Atrial Low‐Voltage Areas in Patients With Persistent Atrial Fibrillation.” Annals of Noninvasive Electrocardiology 22, no. 4: 421. 10.1111/anec.12421. PMC6931520 28090710

